# Family issues and family functioning of Japanese outpatients with type 2 diabetes: a cross-sectional study

**DOI:** 10.1186/1751-0759-7-13

**Published:** 2013-06-25

**Authors:** Hiroaki Takenaka, Juichi Sato, Tomio Suzuki, Nobutaro Ban

**Affiliations:** 1 Takenaka Clinic, 3-16-19 Nippombashi-Higashi, Naniwa-Ku, Osaka 556-0006, Japan; 2 Department of General Medicine, Nagoya University Hospital, 65 Tsurumai-Cho, Showa-Ku, Nagoya 466-0065, Japan

**Keywords:** Family, Family Research, Diabetes Mellitus, Family Relations, Family Characteristics, Family Members

## Abstract

**Background:**

Previous studies confirmed that the control of diabetes is related to family functioning, but the validity of the tools used to assess family functioning in these studies is questionable. Few studies have focused on family issues. In this study, we used a new assessment tool to evaluate family functioning and family issues of patients with type 2 diabetes.

**Methods:**

A cross-sectional questionnaire was given to outpatients with type 2 diabetes at a community hospital in Aichi, Japan, between August 2001 and March 2002. First, the patients were asked to answer FACESKGIV-16, which measures cohesion and adaptability, questions regarding family issues, daily lifestyle, and HAD. Physical and serological data were measured. Family functioning, family issues, and relationships between each parameter and family functioning or family issues were analyzed.

**Results:**

Of the 133 participants, 121 (33.3%) had some sort of family issue. Family issues included “Health problems of family members” (40.9%), “Family life cycle issues” (22.7%), and others.

The best fit multiple regression model (Adjusted R^2^: 0.494, p = 0.020) included Plasma Glucose as an independent variable, and the squared value of cohesion score, depression score of HAD, Total calorie intake, Exercise time, Housekeeping time, and BMI were dependent variables. The results show that extremes of family cohesion with either too many or too few issues related to family functioning are correlated with the plasma glucose level.

**Conclusions:**

Family issues were common among patients with type 2 diabetes, and the extremes of family cohesion were associated with the glucose level, in contrast to the common wisdom that a well balanced family leads to good control of diabetes.

## Introduction

Type 2 diabetes is one of the most common diseases in Japan, affecting approximately 7 million Japanese. Many clinicians believe that diabetic control is related family functioning, and some studies have supported it. But, the validity of the assessment tools used in those studies is questionable. In addition, few studies have focused on the family issues of diabetic patients.

With regard to family issues, other than our two studies, no one has studied them deeply in a single clinical setting or disease, although many researchers to date have studied the family issues of a single family issue. We previously investigated family issues in two clinical settings in Japan. At a general medicine outpatient clinic in a medical school hospital, 30.4% of 250 patients were found to have family issues [1]. The types of their family issues were as follows: 31.6% had members of their family with health problems, 22.4% had family lifecycle issues, and others. At a surgical outpatient clinic in a 60-bed community hospital, 32.1% of 135 patients had family issues [2]. The types of family issues were as follows: 34.3% had members of their family with health problems, 28.6% had family lifecycle issues, and others [2]. The results were similar, but the patients in these studies had a variety of health problems, and the number of patients was too small in each disease category to analyze. The first objective of this research was to determine the frequency and types of family issues in type 2 diabetic outpatients.

With regard to family functioning, previous studies have shown that glycemic control is related family functioning [3-14]. These results are shown as Tables 1 and 2. In these studies, five assessment tools were used: Family Environment Scale (FES) [15,16] (5 studies) [5,9,11-13], Family Adaptability and Cohesion Evaluation Scale III (FACES III) [17] (4 studies) [3,7,9,14], Family APGAR [18] (3 studies) [3,6,8], FAD (Family Assessment Device) [19] (1 study) [10], and Family Adaptability and Cohesion Evaluation Scale at Kwansei Gakuin IV (FACESKG IV) [20,21] (1 study) [4]. Two studies used two tools [3,9]. These family assessment tools have a variety of weak points. The FES and the FAD do not contain all sub-criteria based on their theoretical models [22-24]. FACES III does not establish curvilinearity [25], important within its theoretical model. Thus, conclusions from the aforementioned studies cannot be validated due to the lack of validity of their assessment tools. On the other hand, the FACESKG series was developed in Japan by S. Tatsuki, with due consideration for the cultural and social background in Japan. In the fourth edition FACESKG IV, Tatsuki and his colleagues have succeeded in identifying curvilinearity [26,27]. As far as we know, only one study of the family functioning of diabetic patients using the FACESKG IV-16 has been reported [4]. In that study, there was no relationship between glycemic control and family functioning. However, the glycosylated hemoglobin A1C (HbA1c) level was not determined at a fixed, predetermined time. In this study, we included patients who had a blood examination scheduled on the day of this survey, used both the blood glucose level and the HbA1c level as measures of glycemic control, and measured family functioning, family issues, and glycemic control at the same time. In addition, because total calorie intake, Body Mass Index (BMI), blood pressure, eating behavior, daily lifestyle (sleeping time, working time, housekeeping time, excise time), and mental status are well known to be related with glycemic control, we also investigated the relationship between these parameters and family functioning or family issues.

**Table 1:** Diabetic control and family function - a review

**Publish years**	**Authors**	**Assessment tools**	**Subjects**	**Content**
1981	Andersen, et al. [5]	FES	Diabetic Adolescent	1.) Well controlled youth reported more cohesion and less conflict among family members.
				2.) More parents of well-controlled youth stated that family members were encouraged to behave independently.
				3.) More patients of poorly controlled adolescents believed that diabetes and negatively affected the children’s personality, physical well being, schooling, and participation in activities away from home.
1987	Cardenas, et, al. [6]	FamilyAPGAR	Diabetic adults	Good family function was found in 92% of patients in good control of their diabetes mellitus, in 66% of those in fair control, and only in 50% of those in poor control.
1990	Lawler, et, al. [7]	FACESIII	Diabetic adolescents	The more disengaged the family system, the worse the diabetic control for the adolescents.
			(Between the age of 15 and 18)	
1993	Konen, et, al. [3]	FACESIII	Diabetic adults	1.) A greater population of adults perceived their family to be disengaged than subjects from families without diabetes.
				2.) Adults with NIDDM in good glycemic control as measured by glycosylated hemoglobin (A1c) levels had lower family cohesion and negative affect than those in poor control.
				3.) Conversely those with IDDM with acceptable glycosylated hemoglobin levels had higher family cohesion, less negative affect.
1993	Yamamoto, et, al [8]	FamilyAPGAR	Type ı diabetic inpatients	The family APGAR score was higher in the good control group than in the group with poor control.
1995	Hanson, et, al [9]	FACESIII, FES	Youth 12–20 years of age with IDDM	Positive family relationships (high family cohesion and low family conflict), with IDDM especially during the first years of illness, indirectly related to good metabolic control (through positive adherence behaviors).
1995	Gowers, et, al [10]	FAD	Diabetic adolescents	There was little association between glycemic control and family functioning whether rated by adolescents or parents.
1997	Kawaguchi, et, al [11]	FES	Type I diabetic adolescents and young adults	1) The better expressiveness was, the better diabetic control became. The phenomenon was more seen for men than for women.
				2) Good family organization made the better self control, duly and effectual Insulin therapy, and better controlled diet therapy. (Japanese article)
1997	Carol Dashiff [12]	FES	Type I diabetic adolescents	1.) Single parent’s family had poorly controlled diabetic adolescents, but higher cohesion made better diabetic control.
				2.) Parent’s independency made better diabetic control.
				3.) The higher mother’s responsibility was, the the worse diabetic control became. (Japanese article)
1998	Trief, et, al [13]	FES	Insulin-required diabetic adults	Family cohesion related to better physical function, but none of the family system measures were significant predictors of HbA1c.
1998	Tubiana, et, al [14]	FACES III	French diabetic children (Between the age of 7 and 13)	1.) More diabetic families than comparison families fell into the categories of disengaged (with low levels of cohesion) and rigid (with low levels of adaptability).
				2.) Family functions were significantly and positively correlated with adherence scores, but not with HbA1c levels.
				3.) Children whose families were characterized as rigidly disengaged had a significantly greater number of hypoglycemia and six times as many episodes of ketoacidosis than other diabetic children.
2001	Ikuta [4]	FACESKG IV	Diabetic adults	1.) The majority of diabetic family were enmeshed family and many diabetic families were flexible family.
				2.) Families of type ı diabetic patient had higher adaptability.
				3.) Enmeshed family had low burden and anxiety

**Table 2:** The physical and mental parameters

**Height**	**162.5 ± 8.3 (cm)**
Weight	62.8 ± 10.8 (kg)
BMI (Body Mass Index)	23.8 ± 3.4
Systolic blood pressure	132.6 ± 17.5 (mm Hg)
Diastolic blood pressure	76.3 ± 11.0 (mm Hg)
Indicated total taking calorie	1692.6 ± 176.9 (kcal)
Plasma glucose	146.9 ± 59.6 (mg/dl)
(Reference value < 110 mg/dl)	
Glycosylated hemoglobin (HbA1c)	7.2 ± 1.2%
(Reference value < 5.8%)	
Anxiety score of HAD	5.7 ± 3.5
Depression score of HAD	6.6 ± 2.9

## Methods

### Design and setting

We conducted a cross-sectional questionnaire survey at the diabetic outpatient clinic of a 219-bed community hospital in Aichi prefecture, Japan, between August 2001 and March 2002. First, the doctor-in-charge briefly explained the study and obtained initial oral consent. Then, the examiner-in-charge explained fully the study protocol in a separate room, and written informed consent was obtained. Next, subjects answered the questionnaire in a private area of the hospital. While the patients completed the questionnaires, the patients could ask questions of the research personnel to ensure that they understood and could completely answer all the questions. With the consent of the subjects, the results of blood pressure, BMI, total calorie intake, and glycemic control levels were retrieved afterwards from the chart.

### Participants

The inclusion criteria of this study were: 1) adult type 2 diabetic outpatient followed at the diabetic outpatient clinic of the hospital, 2) provided informed consent, and 3) had a blood examination scheduled on the day of the survey. Patients were excluded who did not want to participate in the study, were living alone without any immediate relatives, were unable to read, write, or communicate, could not understand the purpose of the study, or were considered to be inappropriate for the study by the physician-in-charge.

### Outcome measurements

**Figure 1:**
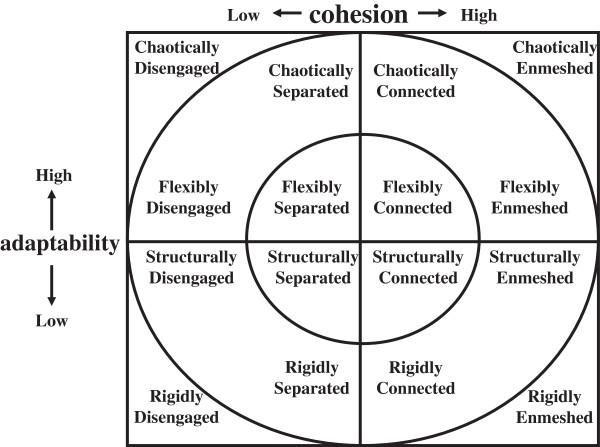
The Circumplex model.

In order to evaluate family issues, we asked “Do you have any worries about your family? If you have, please tell us about them. You are free to decline to answer”. To evaluate family functioning, we used the cohesion and adaptability scores of the FACESKG IV-16. The FACESKG IV is based on the Circumplex model (Figure 1), which is a two-dimensional family function model that relies on a balance between the two dimensions and an avoidance of extremes. Its two dimensions are “cohesion” and “adaptability”. Cohesion indicates the family's emotional bonds. It has four levels: "enmeshed" is the most extreme level (too much closeness), "connected" represents moderate to high closeness, “separated” represents low to moderate closeness, and “disengaged” represents a level of too little closeness. The “connected” and “separated” levels of cohesion are considered to be balanced, and the “enmeshed” and “disengaged” levels are considered to be extreme. Adaptability is the ability of family to adapt to various stressors. Adaptability also has four levels: "chaotic" is the most extreme level (too much change), “flexible” represents high to moderate change, “structured” represents moderate to low change, and “rigid” represents too little change. The "flexible" and “structured” levels of adaptability are considered to be balanced, and the "chaotic" and "rigid" levels are considered to be extreme. The FACESKG IV-16 [28] is a 16-item Thurston scale, a shorter version of the FACESKG IV series. It is especially useful and practical in primary care and family practice settings for its succinctness and ease of use. Its results are based on the sum of the values obtained by multiplying the points for each question by an appropriate coefficient suited to the content. Cohesion and adaptability are each given a score of between −8 points and +8 points, and intermediate scores from −2 to +2 are judged as corresponding to high levels of family functioning. Thus, both extremes in which either too much or too little change on the adaptability axis or too much or too little closeness on the cohesion axis are judged to have a low level of family functioning. It was necessary to adopt the squared value of cohesion score (square of cohesion) to evaluate dysfunction of cohesion. In the same way, it was necessary to adopt the squared value of adaptability score (square of adaptability) to evaluate functional disorder of adaptability.

Physical and serological data were obtained on the same day. We used plasma glucose levels and the HbA1c value of JDS (Japan Diabetes Society) as measures of glycemic control. BMI and total calorie intake were retrieved from the patient’s chart. Daily lifestyle (sleeping time, working time, housekeeping time, and exercise time) was measured by questionnaire. Mental status was measured by HAD (Hospital Anxiety and Depression scale) [29]. HAD is a 14-item, 4-point, self-reported scale for evaluating anxiety and depressive states, consisting of 7 anxiety items and 7 depression items. If each total score is between 0 and 7, the patient has neither anxiety nor depression. If each total score is between 8 and 10, it is doubtful that the patient has anxiety or depression. If each total score is between 11 and 21, the patient, by definition, has anxiety or depression according to the scale. For this investigation, we used the anxiety and depression scores of the HAD Japanese version [30,31].

### Analysis

First, we calculated the percentage of diabetic patients with family issues and categorized the family issues. Next, we analyzed family functioning according to the score of FACESKG IV-16. Then, we attempted to find relationships between family functioning and each parameter by multiple regression analysis (Dependent variables were Plasma Glucose or HbA1c) of the data. Then, we compared each parameter between patients with and without family issues using the Mann–Whitney U-test. We used the SPSS 11.0 for WINDOWS for the analysis in this study.

### Ethical considerations

Written informed consent was obtained from all subjects. The Institutional Review Board of Nagoya University approved the study protocol.

## Results

### Demographic data

Of the 133 outpatients recruited as subjects, 121 fulfilled the inclusion criteria (response rate was 91.0%). Among the 12 subjects excluded, Five did not have sufficient time to become involved in the survey, Three declined to answer the questionnaire, Two lived alone (i.e., no family members), One was unable to write without glasses, and one was unable to communicate with us. One patient did not answer the questions on family issues. Regarding family issues, the response rate was 90.2%. All values in the text, tables, and figures are expressed as means ± standard deviation (SD). The male: female ratio was 91:30 and the average age was 52.1 ± 9.2 years old. The number of family members living with patients was 2.5 ± 1.4 and their working hours were 8.0 ± 2.9 hr. /day and 5.3 ± 1.2 days/week. Physical and mental parameters are presented in Table 2.

### Family issues and parameters

Forty of the 120 patients (33.3%) had family issues. Three had multiple family issues (One had Three issues and two had two issues), giving a total number of family issues of 44. The data on family issues are presented in Additional file 1: Table S1. Almost two-thirds (63.6%) of the 44 family issues were “Health problems of family members” and “Family life cycle issues” such as Aging, Going on to school, Family death, and Child birth.

As a result of Mann–Whitney U test, family issues did not correlate with plasma glucose level (p = 0.245) or HbA1c (p = 0.256). With regard to each issue, “Family life cycle issues” was correlated with patient anxiety (p = 0.004) and depression (p = 0.032). “Divorce” was also correlated patient anxiety (p = 0.039).

### Family functioning and parameters

**Figure 2:**
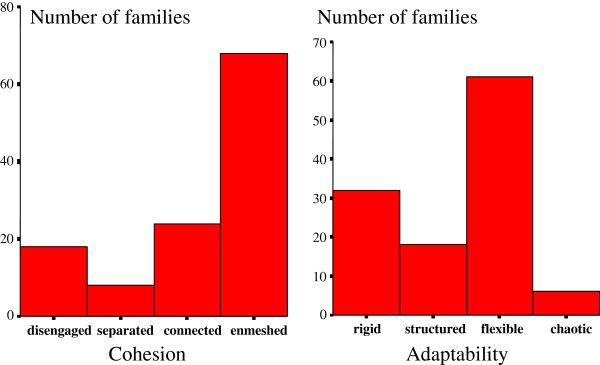
Number of families categorized by family functions (cohesion and adaptability).

The data on family functioning are presented in Figure 2, and the results of the multiple regression analysis with the backward elimination method are presented as Tables 3 and 4. With regard to models containing plasma glucose, the adjusted R^2^ value of the best fit model was 0.494 and the F value was 3.926 (p = 0.020). With regard to models containing HbA1c, the dependent variables of the best fit model were the squared value of the cohesion score and excise time. The adjusted R^2^ value was 0.082, and the F value was 3.859 (p = 0.026). The results of the best fit model identified Plasma Glucose as an independent variable and the squared value of the cohesion score, depression sore of HAD, Total calorie intake, Excise time, Housekeeping time, and BMI as dependent variables.

**Table 3:** The results of the multiple regression analysis (Dependent value was plasma glucose level)

**Model**	**Independent valuables**	**Adjusted R**^**2**^	**F value**	**p (Model)**	**B**	**p (coefficient)**
1	Square value of cohesion	0.348	2.067	0.147	−0.506	0.048*
	Square value of Adaptability				0.08	0.777
	Anxiety				0.909	.079
	Depression				0.132	0.577
	Total calorie intake				0.482	0.054
	Excise time				0.265	0.297
	House keeping time				0.512	0.122
	Sleeping time				0.058	0.87
	BM				−0.212	0.484
2	Square value of cohesion	0.408	2.548	0.084	−0.521	0.030*
	Anxiety				0.108	0.733
	Depression				0.14	0.532
	Total calorie intake				0.487	0.040*
	Excise time				0.263	0.275
	House keeping time				0.48	0.102
	Sleeping time				0.121	0.648
	BM				−0.207	0.47
3	Square value of cohesion	0.455	3.145	0.044*	−0.493	0.020*
	Depression				0.175	0.365
	Total calorie intake				0.51	0.020*
	Excise time				0.299	0.154
	House keeping time				0.469	0.092
	Sleeping time				0.086	0.713
	BM				−0.238	0.362
4	Square value of cohesion	0.494	3.926	0.021*	−0.487	0.016
	Depression				0.17	0.359
	Total calorie intake				0.51	0.016*
	Excise time				0.28	0.15
	House keeping time				0.525	0.022*
	BM				−0.279	0.223

**Table 4:** The results of the multiple regression analysis (Dependent value wasHbA1c value of JDS)

**Model**	**Independent valuables**	**Adjusted R2**	**F value**	**p (Model)**	**B**	**p (coefficient)**
1	Square value of cohesion	0.045	1.355	0.238	−0.167	0.201
	Square value of Adaptability				−0.085	0.515
	BM I				0.111	0.449
	Anxiety				0.175	0.212
	Depression				−0.051	0.733
	Sleeping time				0.162	0.229
	House keeping time				−0.141	0.354
	Excise time				−0.344	0.015*
2	Square value of cohesion	0.072	2.216	0.078	−0.15	0.224
	BM I				0.074	0.55
	Anxiety				0.11	0.374
	Excise time				−0.299	0.017*
3	Square value of cohesion	0.082	2.865	0.044*	−0.142	0.244
	Anxiety				0.118	0.336
	Excise time				−0.298	0.017
4	Square value of cohesion	0.082	3.859	0.026*	−0.141	0.243
	Excise time				−0.309	0.012

Because of missing values, we could not utilize the stepwise analysis, so we utilized the backward elimination method.

## Discussions

Prior to this study, we and previous studies regarded balanced family functioning as being a contributory factor to good control of diabetes, but the findings from this study were in direct contradiction to the theory. They showed through multiple regression analysis that extreme (unbalanced) family cohesion with either too much (enmeshed) or too little (disengaged) functioning was correlated with plasma glucose levels (*p* < 0.05). This implies that too little (disengaged) family cohesion leads to better control of diabetes in some families, but in other families, too much (enmeshed) family cohesion leads to better control of diabetes. Asking the family to collaborate with the patient may be effective in some cases, but has an adverse effect in other cases. As a result, no all-round approach methods can be recommended for families with patients with diabetes, and each case must be decided on its own merits. One major problem is to discern what kind of family functioning is ideal for each family. The discernment will becomes easier if parameters that measure family power to support treatment are developed in future. The mental status of the patient may help in this respect. We used HAD for assessing the mental status of patients with diabetes in this study, but this allows us to understand only depression and anxiety. Other aspects of mental status such as self-efficacy, personality, and others may also affect family function.

Forty of the 120 patients with diabetes (33.3%) had 44 family issues. Almost two-thirds (63.6%) of the family issues were “Health problems of family members” and “Family life cycle issues.” This result was similar to those of two previous studies, one performed at a medical school hospital [1] and another in a surgical department of a community hospital [2], which suggests that 1) family issues are common for patients with chronic diseases and 2) there is no discernible difference across different outpatient settings and different medical problems. Regarding acute medical problems, we did not investigate the family issues and family functioning of patients who were not in a suitable condition to answer questionnaires.

There were also other limitations to this research, some being common to research projects on the family, while others were specific to this project. In this study, our measurement of family functioning was estimated only by the patients themselves: other family members may have had different views. However, the measurement of family functioning is difficult because it is not known whether averaging the score of various family members accurately indicates family functioning. Such limitations are common in research projects on the family and are related to a lack of gold standard criteria for the assessment of family dysfunction and definitions of family issues. With regard to limitations specific to this study, beside cultural bias, male subjects outnumbered female subjects, and the small sample size might have affected the results. In addition, we did not disaggregate our findings on the basis of the length of illness (diabetes mellitus). With regard to the measure of glycemic control, HbA1c is a better indicator of long-term glycemic control than the blood glucose level, as it reflects levels over the previous one month. So, we think the models using HbA1c were worse in our study. Thus, in future studies, it would be best to ask patients about family functioning and family issues and to obtain a blood sample for evaluation of HbA1c one month later. It is well-known that medication decreases plasma glucose levels even if patients with hyperglycemia exhibit family dysfunction and/or family issues. A study on patients with diabetes treated only through dietary and exercise therapy would eliminate this confounder. We expect subsequent studies to overcome these limitations and biases, and to include the tools, location, male: female ratio, and effects of medications in order to achieve clearer results and develop treatments for application within the clinical setting.

## Conclusions

In this study, 40 of the 120 patients (33.3%) had family issues. Almost two-thirds (63.6%) of the family issues were “Health problems” and “Family life cycle issues.” “Family life cycle issues” and “Generation conflict” were related to patient anxiety (*p* < 0.01).

Extreme family cohesion (too much or too little closeness) was correlated with plasma glucose level (*p* < 0.05), in contrast to the common wisdom that balanced family functioning leads to good control of diabetes. Higher family cohesion contributed to lower anxiety (*p* < 0.05).

Family issues were common among the patients with diabetes, and they were not disease-specific issues.

There is no all-round approach for the families of patients with diabetes; so, we need to formulate a better approach on an individual family basis.

## Abbreviations

FACESKG IV: Family Adaptability and Cohesion Evaluation Scale at Kwansei Gakuin IV; HAD: Hospital Anxiety and Depression scale; FES: Family Environmental Scale; FACES III: Family Adaptability and Cohesion Evaluation Scale III; FAD: Family Assessment Device; ASO: Arteriosclerosis obliterans; HbA1c: Hemoglobin A1c (Glycosylated Hemoglobin); SD: Standard deviation; BMI: Body Mass Index; SBP: Systolic Blood Pressure.

## Competing interests

The authors declare that they have no competing interests.

## Authors’ contributions and background information

HT is the corresponding author. He wrote the paper, analyzed the data, and contributed to written informed consent and the discussion. He has been Vice president of Takenaka Clinic in Osaka, Japan since 1998 and Chair of the Kansai Society of Family Practice since 2006. His primary research interests focus on family and community approaches to family medicine. JS was the doctor in charge of some of the patients. He gathered data, contributed to ethical considerations, oral informed consent, and the discussion. He is Assistant Professor and Associate Chair of the Department of General Medicine at Nagoya University Hospital. He took his current position in 1999. His primary research interests focus on preventive medicine in primary health care. TS was the doctor in charge of the other patients. He gathered data, contributed to oral informed consent, and the discussion. He is Assistant Professor and Associate Chair of the Department of General Medicine at Nagoya University Hospital. He took his current position on 2000. His primary research interests focus on medical education and diagnosis in primary health care. NB contributed to the discussion, edited the paper and helped with translation. He is Professor and Chair of the Department of General Medicine at Nagoya University Hospital. He has been in his current position since 1998. His primary research interests focus on medical education and primary health care. All authors read and approved the final manuscript.

## Supplementary Material

Additional file 1: Table S1 Family issues with the diabetic patients. Click here for file

## Prior presentation

(Regarding Family Issues) 15th KINKI Regional conference of the Japanese Academy of Primary Care, Oct 21, 2001, WAKAYAMA, JAPAN.

(Regarding Family Functioning) 7th Japanese Academy of Diabetic Education and Nursing, Oct 6, 2002, NAGOYA, JAPAN.
