# Supplementary material for: Family issues and family functioning of Japanese outpatients with type 2 diabetes: a cross-sectional study
Source: Biopsychosoc Med. 2013 Jun 25;7:13. doi: 10.1186/1751-0759-7-13 (PMC3700776; doi:10.1186/1751-0759-7-13)
Supplement: Additional file 1: Table S1 — Family issues with the diabetic patients. [file 1751-0759-7-13-S1.ppt]

## Slide 1
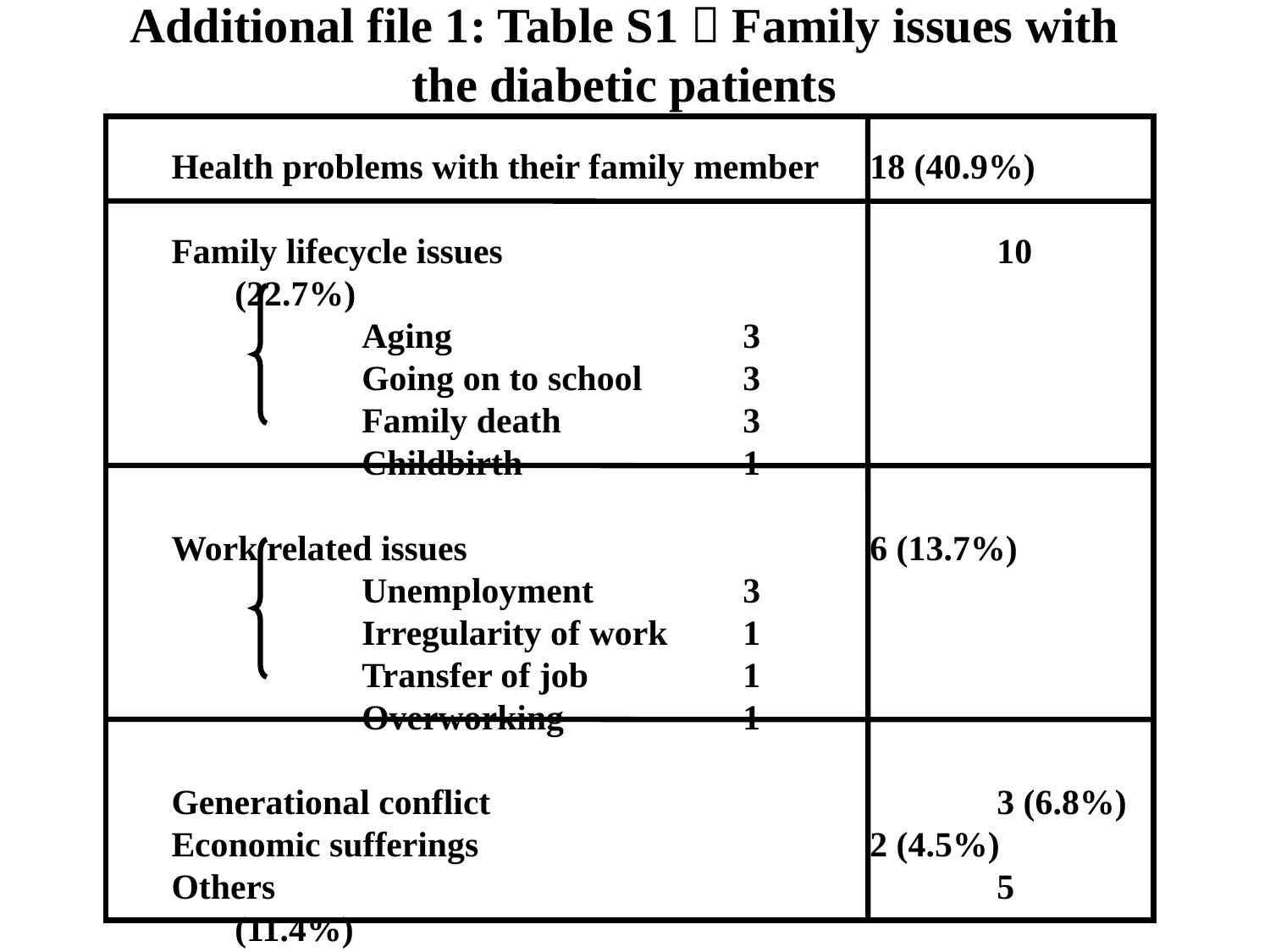

# Additional file 1: Table S1：Family issues with the diabetic patients
Health problems with their family member 	18 (40.9%)
Family lifecycle issues				10 (22.7%)
		Aging			3
		Going on to school	3
		Family death		3
		Childbirth		1
Work related issues				6 (13.7%)
		Unemployment		3
		Irregularity of work	1
		Transfer of job		1
		Overworking		1
Generational conflict				3 (6.8%)
Economic sufferings				2 (4.5%)
Others						5 (11.4%)
（divorce, don’t want to answer, and so on）
